# Biogenic Magnetosomes as Magnetically Recoverable Catalysts for Benzyl Alcohol Oxidation

**DOI:** 10.1021/acsomega.6c07960

**Published:** 2026-10-09

**Authors:** Josh Bond, Marcia Mendes de Carvalho, Amin Osatiashtiani, Tim William Overton, Oliver Brooks, Vesna Najdanovic-Visak, Alfred Fernandez-Castane

**Affiliations:** † Energy and Bioproducts Research Institute, 1722 Aston University, Aston Triangle, Birmingham B4 7ET, United Kingdom; ‡ Aston Institute for Membrane Excellence, Aston University, Aston Triangle, Birmingham B4 7ET, United Kingdom; § School of Chemical and Process Engineering, University of Leeds, Leeds LS2 9JT, United Kingdom; ∥ School of Chemical Engineering, 1724 University of Birmingham, Edgbaston, Birmingham B15 2TT, United Kingdom; ⊥ School of Metallurgy and Materials, University of Birmingham, Edgbaston, Birmingham B15 2TT, United Kingdom

## Abstract

Biogenic magnetosomes are intracellular single-domain magnetic nanoparticles (MNPs) produced by *Magnetospirillum gryphiswaldense* MSR1, consisting of crystalline magnetite (Fe_3_O_4_) encapsulated within an organic membrane. Despite their controlled structure and widespread study in biomedical and magnetic applications, their use in catalysis has not been explored. Here, magnetosomes are demonstrated for the first time as heterogeneous catalysts for benzyl alcohol oxidation using hydrogen peroxide. Under aqueous batch conditions, catalytic performance was tested, varying catalyst loading and temperature. Among the loadings tested, the highest conversion was achieved using 5.2 mol % Fe_3_O_4_ equivalent, reaching 64 ± 4% after 24 h with 36 ± 5% benzaldehyde selectivity. At approximately matched Fe_3_O_4_ contents, this performance was broadly comparable to commercial Fe_3_O_4_, which produced 72 ± 2% conversion and 38 ± 3% selectivity. Temperature screening of magnetosome catalysts increased conversion up to 75 ± 11% at 85 °C with 30 ± 4% benzaldehyde selectivity. Meanwhile milder conditions improved selectivity to 43 ± 2% at the expense of lower conversion, reflecting the expected conversion/selectivity trade-offs seen in wider iron oxide-catalyzed peroxide systems. Catalytic recyclability was demonstrated over five reaction cycles, where magnetic recovery enabled the spent mixture to be removed and replaced without centrifugation or filtration. During these cycles conversion decreased only modestly from 71 ± 5% to 65 ± 4% with selectivity remaining between 33 and 38%. Overall, this work demonstrates that biogenic magnetosomes can function as catalytic materials and expands the potential applications of magnetotactic bacteria-derived nanostructures.

## Graphical abstract

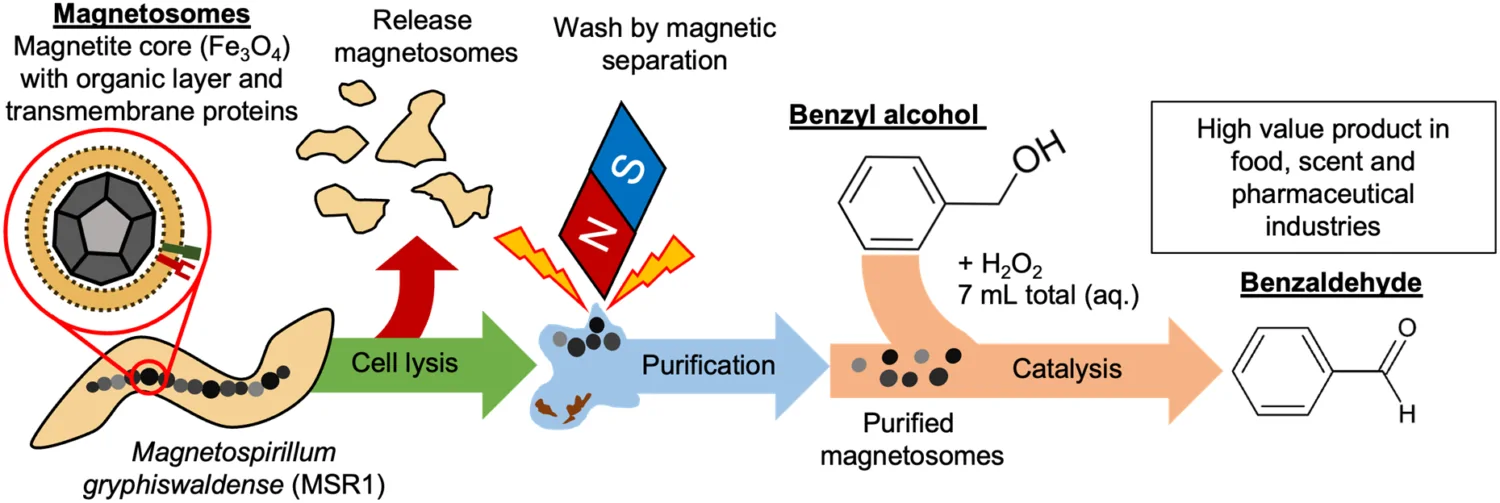

## 1 Introduction

Oxidation of primary alcohols to aldehydes is a fundamental industrial transformation.^1,2^ Benzyl alcohol oxidation is particularly relevant because benzaldehyde is widely used in flavor, fragrance, cosmetic, pharmaceutical and fine chemical manufacturing.^3,4^ Conventional routes such as toluene oxidation or benzyl chloride hydrolysis can generate chlorine-containing impurities and problematic waste, motivating cleaner approaches.^4,5^

Achieving high conversion while suppressing overoxidation to carboxylic acids remains a central challenge in alcohol oxidation, particularly given the historical reliance on stoichiometric heavy metal oxidants that generate hazardous waste streams.^6,7^ The transition toward greener oxidants, particularly hydrogen peroxide, has increased the demand for efficient heterogeneous catalysts capable of delivering acceptable activity and selectivity under milder and more sustainable conditions.^8^ Although noble metal catalysts can deliver high activity, their cost and sustainability limitations have driven interest toward abundant, low-toxicity iron-based alternatives.^1,7,9^ Consequently, attention has shifted toward magnetically recoverable iron-based catalysts operating under milder, environmentally friendly conditions.^10–12^ MNPs have been widely employed as heterogeneous catalysts for selective alcohol oxidation, particularly in systems designed for magnetic recovery.^1,7,8,12–15^ In particular, iron oxide-based MNPs at low loadings, combined with hydrogen peroxide in acetonitrile, have demonstrated efficient oxidation of benzyl alcohol to benzaldehyde, with water as the sole byproduct.^16,17^ However, for many iron oxide and iron-based catalyst systems (Table 1), balancing conversion, selectivity and catalyst recovery remains challenging.^1,2,6–8,12–14,17,18^

**Table 1:** Catalysed Selective Oxidation Reactions of a Range of Alcohols to Corresponding Aldehydes

Catalyst	Temp °C	Time	Solvent	Oxidiser	Substrates	Products	Conversion (%)	Selectivity (%)	refs
Fe/MCM-41	Microwave 70	48h	Acetonitrile	H_2_O_2_	Benzyl alcohol	Benzaldehyde	25	>95	^17^
Au–Pd/TiO_2_	120	1h	Solvent-free	O_2_	Benzyl alcohol	Benzaldehyde	88	67	^18^
Pt black-SiO_2_	90	>120h	*t*-amylOH	H_2_O_2_	(*E*)-cinnamyl alcohol	(*E*)-cinnamaldehyde	100	97	^6^
Ag–Co/S	75	80 min	Ethanol	O_2_	cinnamyl alcohol	cinnamaldehyde	90	99	^2^
MnFe_2_O_4_	Microwave 120	2 h	Solvent-free	*t*-BuOOH	Benzyl alcohol	Benzaldehyde	79.1	>99	^8^
Fe_3_O_4_ (magnetite)							51.2	>99	
CoFe_2_O_4_							88.9	>99	
NiFe_2_O_4_							46.2	>99	
CuFe_2_O_4_							86.7	>99	
ZnFe_2_O_4_							44.7	>99	
Fe/Al-SBA-15	120	1 h	*n*-heptane	Pressurised O_2_	Benzyl alcohol	Benzaldehyde	98	95	^1^
Fe/Al-SBA-15	80	3 min	Acetonitrile	H_2_O_2_	4-nitrobenzyl alcohol	4-nitrobenzaldehyde	88	99	^7^
		15 min					95	99	
		3 min			3-methoxy benzyl alcohol	3-methoxybenzaldehyde	53	93	
		30 min					97	90	
		3 min			6-methyl heptanol	6-methylheptanal	15	63	
		15 min					45	68	
Nano-Fe_3_O_4_	75	12 h	Solvent free	H_2_O_2_	Benzyl alcohol	Benzaldehyde	33	97	^14^

Nanoparticle catalysts are attractive because their high surface-area-to-mass ratios can enhance reactivity.^8^ MNPs are particularly relevant due to their abundance, low toxicity, compatibility with H_2_O_2_-driven oxidation, and magnetic recoverability, enabling catalyst separation and reuse without filtration or centrifugation.^14,19–21^ However, conventional chemically and physically synthesized iron oxide often requires elevated temperatures and can aggregate, requiring stabilization or immobilization.^22^ Since catalytic performance depends strongly on phase, morphology, size distribution and surface chemistry, poor control over aggregation can limit reproducibility.^23^ Biogenic iron oxide-based MNPs produced by magnetotactic bacteria (MTB) offer an alternative, produced under mild conditions, with biomineralisation providing control over particle size, morphology and organization, while organic components may improve stability and reduce aggregation.^24^

Magnetosomes are membrane-bound intracellular MNPs, composed of magnetite (Fe_3_O_4_) or greigite (Fe_3_S_4_), produced by MTB.^25–27^ Magnetosomes are formed through tightly regulated biomineralisation under mild aqueous conditions. This yields highly crystalline particles with narrow size distribution (20–120 nm), consistent morphology, typically arranged into ferrimagnetic intracellular chains, including in the model MTB *Magnetospirillum gryphiswaldense* (MSR1).^28–31^ This level of structural control is difficult to reproduce consistently using conventional precipitation-based synthesis.^22,30^ Each magnetosome is enclosed by a native phospholipid membrane containing specialized proteins, which improves colloidal stability, reduces aggregation, and provides potential anchoring points for further functionalization.^25,26,28,31,32^ Following cell disruption, magnetosomes can be purified by magnetic separation, enabling their use as isolated functional nanomaterials.^33^ This has supported investigation into biomedical and environmental applications, including imaging, drug delivery and wastewater bioremediation.^32,34–38^

However, despite their intrinsic magnetic recoverability and controlled crystal morphology, their use as heterogeneous oxidation catalysts remains unexplored. Accordingly, this study evaluates purified magnetosomes from MSR1 for aqueous benzyl alcohol oxidation using hydrogen peroxide, providing a proof-of-concept demonstration of their catalytic activity as biogenic MNPs.

## 2 Materials and Methods

### 2.1 Chemicals

All chemicals were purchased from Sigma-Aldrich or Thermo Fisher and were ≥99% pure unless stated otherwise. For catalyst preparation, *Magnetospirillum gryphiswaldense* MSR1 was obtained from DSMZ (Germany) and grown in flask standard medium (FSM) containing soybean peptone (3 g/L), yeast extract (0.1 g/L), NaNO_3_ (0.34 g/L), KH_2_PO_4_ (0.1 g/L), HEPES (2.38 g/L), MgSO_4_·7H_2_O (0.15 g/L), potassium l-lactate (60% w/v; 5.83 mL/L), Fe­(III) citrate (100 mM; 1 mL/L), and EDTA-chelated trace elements solution (5 mL/L; pH 6.5). The EDTA-chelated trace-element solution comprised EDTA disodium salt (5.2 g/L), FeSO_4_·7H_2_O (2.1 g/L), H_3_BO_3_ (30 mg/L), MnSO_4_·H_2_O (85.4 mg/L), CoCl_2,_ (190 mg/L), NiCl_2_·6H_2_O (4 mg/L), CuCl_2_·2H_2_O (2 mg/L), ZnSO_4_·7H_2_O, (44 mg/L) and Na_2_MoO_4_·2H_2_O (36 mg/L). Growth media was adjusted to pH 7 using 1 M NaOH and autoclaved at 121 °C for 20 min.^39^ For catalytic testing and HPLC analysis, benzyl alcohol, H_2_O_2_ (12% w/v), and benzaldehyde were used. Milli-Q water was used throughout. For catalyst characterization, purified magnetosomes were benchmarked against commercial Fe_3_O_4_ powder (Inoxia; CAS: 1309–38–2).

#### 2.1.1 Production of *Magnetosomes*

*Magnetospirillum gryphiswaldense* MSR1 precultures were grown in FSM and used to inoculate a 5 L stirred-tank bioreactor (Biostat B, Sartorius UK Ltd., UK) to an initial optical density of OD_565_ ≈ 0.05. The bioreactor was operated at 30 °C with an agitation speed of 150 rpm. Dissolved oxygen (pO_2_) was controlled at 0.1% with air supplied through a sparger, and pH maintained at 7.0 via auto addition of 1 M HCl. Cultures were grown for 72 h (1–1.5 OD_565_). Wet cell pellets were harvested by centrifugation and stored at −80 °C.^28,40^

#### 2.1.2 Magnetosome Isolation and Purification

Magnetosome purification was performed using an established methodology.^40^ Briefly, frozen cells were resuspended in disruption buffer (50 mM HEPES, 4 mM EDTA, pH 7.4) and lysed through a cell disruptor operated at 10 kpsi (X2 Pro, Constant systems Ltd., UK). Magnetosomes were collected using an NdFeB N45 permanent magnet with a nominal maximum flux density of 1.4 T. The supernatant containing nonmagnetic cellular debris was removed, and the magnetically retained material was resuspended and washed 10 times in fresh disruption buffer. Following washing, magnetosomes were resuspended as 6 × 1 mL Milli-Q aliquots in Ti 70.1 ultracentrifuge tubes layered atop a 5 mL 60% (w/v) sucrose cushion. Samples were centrifuged at 45,000 rpm for 2.5 h at 4 °C (Optima XPN-100 with Ti 70.1, Beckman Coulter, USA). The supernatant was discarded, and magnetosomes were resuspended in Milli-Q water to 6 mL and stored at −80 °C overnight. Subsequently, samples were freeze-dried for at least 24 h using a benchtop freeze-drier (Labylo, Frozen Time Ltd., UK; −55 °C cold trap, 1 mbar), producing a dry magnetosome powder that was stored in a silica desiccator until catalytic use.

### 2.2 Catalyst Characterization

#### 2.2.1 Thermogravimetric Analysis (TGA)

TGA was performed under a nitrogen flow of 40 mL/min (DSC 2 STARe, Mettler Toledo, USA). Commercial magnetite (Fe_3_O_4_) and purified magnetosomes were analyzed in triplicate by heating from 40 to 900 °C at a rate of 10 °C/min.

#### 2.2.2 X-ray Diffraction (XRD)

XRD patterns were collected for commercial Fe_3_O_4_ and purified magnetosomes using a diffractometer (D8 Advance, Bruker, USA) equipped with a LynxEye position-sensitive detector and Cu Kα radiation. Data were recorded over a 2θ range of 10–80°, using a step size of 0.04° and a counting time of 1 s per step.

#### 2.2.3 Magnetometry Measurements

Magnetic properties were determined at room temperature using a vibrating sample magnetometer (VSM; Lake Shore 8600 Series, Lake Shore Cryotronics, USA). Approximately 50 mg each of dried magnetosomes and dried commercial Fe_3_O_4_ were analyzed. Hysteresis loops were obtained by saturating the sample in a large positive magnetic field (+500 mT). The field was then decreased to a reversal field (−500 mT). Saturation magnetic polarization (*J*_s_), remanent magnetic polarization (*J*_r_), and coercivity (*H*_c_) were determined after the high-slope paramagnetic correction, and the ratio of remanence to saturation magnetization (*J*_r_/*J*_s_) was calculated.

#### 2.2.4 Transmission Electron Microscopy (TEM)

MSR1 samples containing magnetosomes were adsorbed onto carbon-coated copper grids. Imaging was performed using a JEOL 2100F FEGTEM operating at 200 kV, equipped with a Gatan K3-IS direct detection camera, JEOL bright field and dark field STEM detectors.

### 2.3 Catalytic Testing of Magnetosomes for Benzyl Alcohol Oxidation

Benzyl alcohol oxidation was performed under heterogeneous batch conditions in sealed 15 mL polypropylene centrifuge tubes. Reactions contained benzyl alcohol, H_2_O_2_ and Milli-Q water (25 mM benzyl alcohol, 0.10% v/v H_2_O_2_, 7 mL total volume; alcohol-to-oxidant molar ratio was 1:1.7 ^41^). Control reactions containing 0 mg catalyst were performed both with and without H_2_O_2_. Commercial Fe_3_O_4_ was evaluated at loadings of 0.5, 2, 10, and 20 mg, while magnetosomes were evaluated at loadings of 2 and 10 mg. Reactions were heated in a thermomixer (HC, Starlab, Switzerland; 750 rpm) at a temperature range of 75, 80, and 85 °C.^7,14^ All catalytic reactions were performed as independent triplicates for each condition.

Catalyst loadings were selected relative to reported values for iron oxide-catalyzed benzyl alcohol oxidation, where typical catalyst-to-substrate ratios fall within 10–50 mg/mmol.^42,43^ Commercial Fe_3_O_4_ was initially screened across a broader loading range to identify an appropriate benchmark condition. Based on TGA, purified magnetosomes yielded 21 wt % inorganic residue, which was treated as Fe_3_O_4_-equivalent for approximate mass normalization rather than as definitive Fe_3_O_4_ content. Accordingly, a 10 mg magnetosome loading was estimated to represent approximately 2.1 mg Fe_3_O_4_ equivalent, corresponding to 5.2 mol % relative to benzyl alcohol. The lower 2 mg magnetosome loading corresponded to approximately 1.0 mol % Fe_3_O_4_ equivalent.

Catalyst reuse was assessed over five consecutive cycles using 10 mg magnetosomes under fixed conditions (80 °C). After each cycle, magnetosomes were immobilized against the reactor wall using a 1.4 T NdFeB N45 permanent magnet, the reaction mixture was removed, and the recovered catalyst was washed with Milli-Q water before resuspension in fresh reaction solution.

The reaction mixtures were cooled to room temperature and analyzed using a high-performance liquid chromatography (HPLC; 1200 Infinity series, Agilent, USA) equipped with a Poroshell 120 EC-C18 column (Agilent, USA). The mobile phase consisted of 40% (v/v) acetonitrile in Milli-Q water, operated at 25 °C with a flow rate of 1.5 mL/min. Benzyl alcohol and benzaldehyde were detected using refractive index and UV diode-array detectors. Calibration curves were generated using standards.

Reactant conversion, product yield, and selectivity were calculated according to eqs 1–3. Where *n* is the number of moles and subscript *i* and *f* denote the initial and final states, respectively.

1 $$\mathrm{Alcohol conversion}\;\%=\frac{n_{alcohol,i}-n_{alcohol,f}}{n_{alcohol,i}}\times 100$$

2 $$\mathrm{Product yield}\;\%=\frac{n_{product,f}}{n_{alcohol,i}}\times 100$$

3 $$\mathrm{Product selectivity}\;\%=\frac{n_{product,f}}{n_{alcohol,i}-n_{alcohol,f}}\times 100$$

### 2.4 Statistical Analysis

Data from catalytic testing is presented as mean ± standard deviation from three biological replicates. Comparisons between commercial Fe_3_O_4_ and magnetosomes were compared using two-sided Welch’s *t*-tests, which assume normality but do not assume equal variances. Temperature-dependent results were analyzed using one-way ANOVA followed by Tukey-adjusted pairwise comparisons, which assume normally distributed residuals and equal variance among groups. Catalyst reuse was analyzed using one-way repeated-measures ANOVA, which assumes normality of the within-subject differences and sphericity, meaning equal variance among these differences. Given the small sample size (*n* = 3), these normality and variance assumptions could not be tested robustly, and the results were interpreted cautiously. Statistical significance was defined as *p* < 0.05. All analyses were performed in Python 3 using SciPy and statsmodels.

## 3 Results and Discussion

### 3.1 Catalyst Characterization

Preliminary magnetosome catalyst characterization enabling comparison with commercial magnetite (Fe_3_O_4_) was performed by TGA, XRD and magnetometry (Figure 1). TGA was used to estimate the Fe_3_O_4_ fraction from residual mass after organic decomposition (Figure 1A), XRD confirmed the crystalline Fe_3_O_4_ phase relative to a commercial reference (Figure 1B), and magnetic hysteresis measurements assessed magnetic functionality and recoverability (Figure 1C).

**Figure 1:**
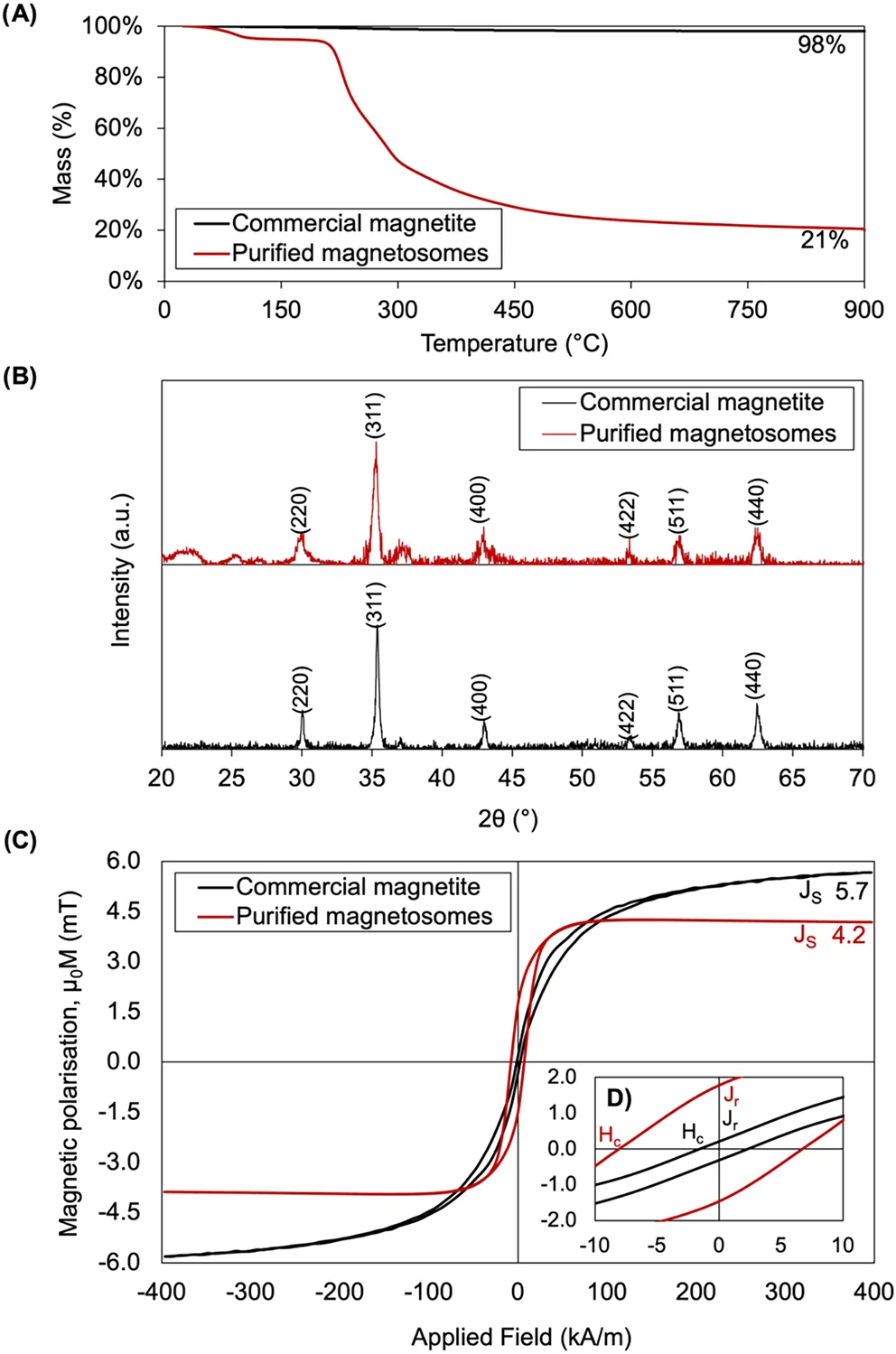
Physicochemical characterization of purified magnetosomes compared with commercial magnetite (Fe_3_O_4_). (A) TGA profiles showing residual mass (%) up to 900 °C for commercial Fe_3_O_4_ and purified magnetosomes (*n* = 3). (B) XRD patterns of commercial Fe_3_O_4_ and purified magnetosomes, showing diffraction intensity as a function of 2θ, with characteristic Fe_3_O_4_ peak positions labeled. (C) Full-field magnetic hysteresis loops of commercial Fe_3_O_4_ and purified magnetosomes, matched by total sample mass and measured under identical conditions, showing the approach to saturation magnetic polarization (*J*_s_). (D) Expanded low-field region of the hysteresis loops, highlighting remanent magnetic polarization (*J*_r_; positive *y*-intercept) and coercive field (*H*_c_; field at which magnetic polarization crosses zero).

Figure 1A shows that commercial Fe_3_O_4_ lost only ∼2% mass up to 900 °C, consistent with removal of physisorbed water.^44^ In contrast, purified magnetosomes showed substantial mass loss from moisture removal and thermal degradation of associated proteins and lipids, giving a final inorganic residue of 21 wt %. Previous TGA analysis of magnetosome material reported 60% mass loss by 380 °C, attributed to the decomposition and desorption of surface-bound proteins.^45^ The stable residue observed here was therefore attributed predominantly to inorganic iron oxide derived from the crystalline magnetosome cores. Furthermore, controlled biomineralization by MSR1 has been shown to produce stoichiometric, structurally pure intracellular crystalline Fe_3_O_4_.^46^ Magnetosome Fe_3_O_4_ can nevertheless undergo oxidation and phase transformation at elevated temperatures under oxidizing conditions.^47^ The composition of the recovered residue was not independently confirmed. The 21 wt % residue was therefore treated only as an Fe_3_O_4_-equivalent value for approximate mass normalization rather than as definitive Fe_3_O_4_ content. This provided a practical basis for comparing similar nominal iron-oxide loadings but did not account for potential differences in surface area, membrane coverage, Fe_3_O_4_ surface accessibility or active-site density between magnetosomes and commercial Fe_3_O_4_.

Figure 1B shows that magnetosome-derived samples retain the characteristic Fe_3_O_4_ reflections seen in commercial Fe_3_O_4_, including the (220), (311), (400), (422), (511), and (440) planes, confirming preservation of the crystalline Fe_3_O_4_ core.^48^ Slight peak broadening likely reflects nanoscale crystallite size and biological sample complexity, although partial oxidation toward maghemite cannot be excluded due to overlapping XRD patterns.^49^ Weak features between 20 and 30° 2θ are attributed to residual biological lipids and proteins.^50,51^

Figure 1C shows higher saturation magnetic polarization for commercial Fe_3_O_4_ (*J*_s_ = 5.7 mT) than magnetosomes (*J*_s_ = 4.2 mT), consistent with dilution by nonmagnetic organic material and the 21 wt % Fe_3_O_4_-residue content determined by TGA.^31,52^ In the low-field region (Figure 1D), magnetosomes showed higher remanent magnetization, coercivity and loop squareness (*J*_r_ = 1.8 mT; *H*_c_ = 9 kA/m; *J*_r_/*J*_s_ = 0.43) than commercial Fe_3_O_4_ (*J*_r_ = 0.3 mT; *H*_c_ = 1.7 kA/m; *J*_r_/*J*_s_ = 0.05). This is consistent with stable single-domain magnetosomes arranged in chains, where dipolar coupling and uniaxial shape anisotropy enhance magnetic retention.^53,54^ As the samples were measured without prior magnetic alignment, the markedly greater loop squareness of the magnetosomes despite random chain orientation further indicates that this anisotropy is intrinsic to the intact chain structure. By contrast, the lower *J*_r_ and *H*_c_ values of commercial Fe_3_O_4_ are consistent with weakly hysteretic behavior reported for synthetic iron oxide nanoparticle systems.^55^ These properties support magnetic recovery, which is advantageous for recyclable heterogeneous catalysis.^54,56^

In addition, TEM imaging was used to confirm catalyst morphology and nanoscale structure. Bright-field imaging showed MSR1 magnetosome chains used for catalyst preparation (Figure 2A), while dark-field imaging resolved individual Fe_3_O_4_ nanocrystals within the chain (Figure 2B). The corresponding particle-size distribution is presented in Figure 2C.

**Figure 2:**
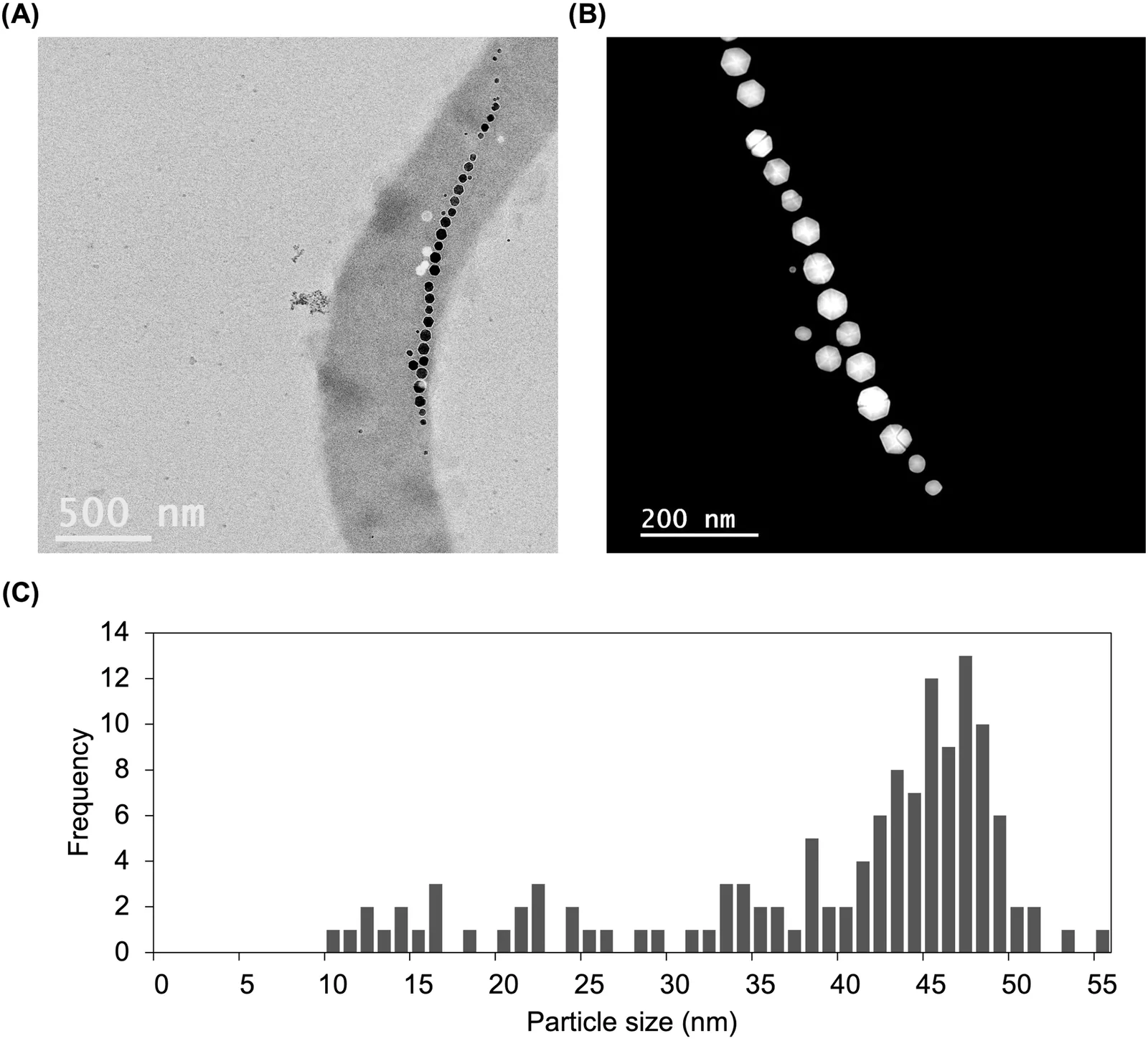
Transmission electron microscopy (TEM) images of magnetosomes in *Magnetospirillum gryphiswaldense* (MSR1) cells. (A) Bright-field TEM image showing an intact MSR1 cell containing a magnetosome chain. (B) Dark-field TEM image of a magnified section of a representative magnetosome chain, highlighting individual magnetite (Fe_3_O_4_) nanocrystals. (C) Particle-size histogram of Fe_3_O_4_ nanocrystals measured from TEM images.

Figure 2B provides a representative example of the dark-field TEM images used for particle-size analysis. Using the same measurement methodology, magnetosome diameters were determined across 10 collected dark-field TEM images. The magnetosomes had an average diameter of 39.0 ± 10.1 nm (*n* = 127), consistent with the typical 35–50 nm size range reported for MSR1 magnetosomes.^57^ The particle-size histogram (Figure 2C) showed a dominant size range of approximately 40–50 nm, consistent with mature MSR1 magnetosomes, while the smaller size clusters at approximately 10–17 and 20–27 nm are consistent with crystals that remained at earlier stages of intracellular biomineralization when the cells were harvested.^58,59^ This distribution supports retention of canonical magnetosome morphology while reflecting the expected size variation within an actively biomineralizing culture. As nanoparticle size and morphology influence heterogeneous catalytic behavior, this consistent biological size control may be relevant to catalyst function.^60^ Together, these analyses establish the phase identity, morphology and magnetic properties required for this initial catalytic assessment. However, BET surface area was not determined as part of this characterization and consequently catalyst mass provided the available basis for subsequent comparison. This limitation is considered further in Section 4.

### 3.2 Catalytic Oxidation of Benzyl Alcohol with Hydrogen Peroxide

Catalytic activity was evaluated under aqueous batch conditions using H_2_O_2_ as the oxidant, with reaction parameters selected to remain within substrate/product solubility limits and reflect literature conditions for iron oxide-catalyzed benzyl alcohol oxidation.^7,41^ Commercial Fe_3_O_4_ was first evaluated across a loading range of 0.5 to 20 mg, alongside 0 mg controls with and without H_2_O_2_, to establish its catalytic activity and identify an appropriate benchmark loading. Magnetosome loading was subsequently normalized on an approximate Fe_3_O_4_-equivalent basis using the 21 wt % inorganic residue determined by TGA (Figure 1A). Accordingly, 10 mg magnetosomes represented an estimated 2.1 mg Fe_3_O_4_ equivalent, providing a nominal mass match to the 2 mg commercial Fe_3_O_4_ benchmark, while 2 mg magnetosomes represented a deliberately under-dosed condition.^42,43^ This comparison should be regarded as approximate and does not constitute a comparison of intrinsic catalytic activity. However, for simplicity catalytic performance was compared at approximately matched Fe_3_O_4_ contents rather than by calculating turnover frequency (TOF). Catalytic performance is summarized in Figure 3, with Figure 3A showing the commercial Fe_3_O_4_ loading screen and control reactions, Figure 3B showing the effects of magnetosome loading and reaction time, and Figure 3C showing the effect of temperature between 75 and 85 °C. The corresponding conversion, selectivity and calculated benzaldehyde yield data are provided in Table S1.

**Figure 3:**
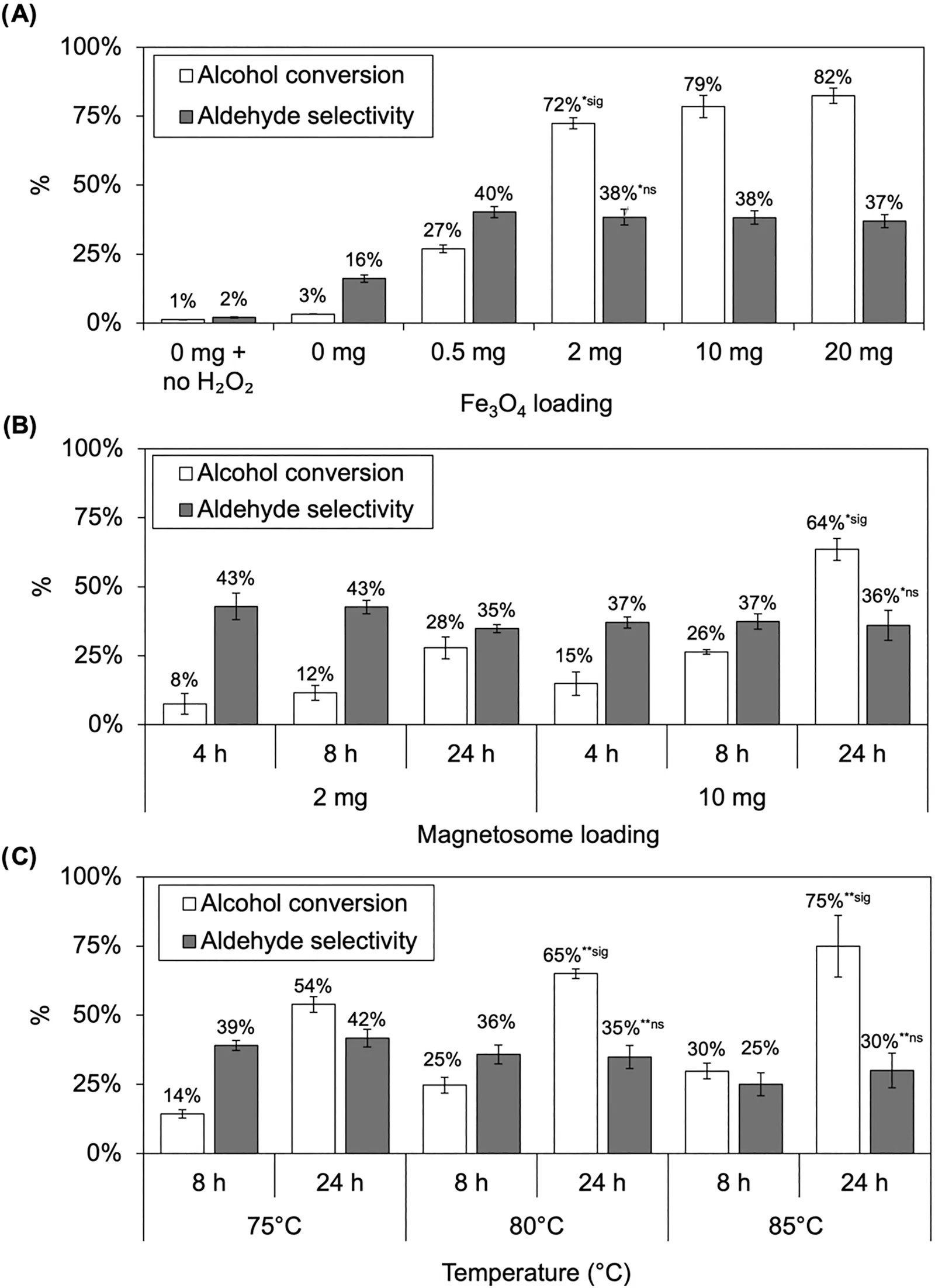
Catalytic oxidation of benzyl alcohol under aqueous reaction conditions. (A) Commercial Fe_3_O_4_ loading screen comprising 0, 0.5, 2, 10, and 20 mg Fe_3_O_4_, corresponding to approximately 0, 1.2, 4.9, 24.7, and 49.4 mol % relative to benzyl alcohol, respectively, including 0 mg controls with and without H_2_O_2_. (B) Effect of magnetosome loading (2 and 10 mg), corresponding to approximately 1.0 and 5.2 mol % Fe_3_O_4_ equivalent relative to benzyl alcohol, respectively. (C) Effect of reaction temperature using 10 mg magnetosomes. Unless otherwise stated, reactions contained 25 mM benzyl alcohol and 0.10% (v/v) H_2_O_2_ (alcohol-to-oxidant molar ratio ≈ 1:1.7) in a total volume of 7 mL adjusted with Milli-Q water and were conducted at 80 °C and 750 rpm for 24 h. The * annotations identify statistical comparisons between 2 mg commercial Fe_3_O_4_ and 10 mg magnetosomes at approximately matched Fe_3_O_4_ contents after 24 h, assessed using two-sided Welch’s *t*-tests: conversion, *p* = 0.006 (significant; sig); selectivity, *p* = 0.171 (not significant; ns). The ** annotations identify Tukey-adjusted pairwise comparisons following one-way ANOVA between magnetosome-catalyzed reactions conducted at 80 and 85 °C for 24 h: conversion, *p* = 0.012 (sig); selectivity, *p* = 0.130 (ns). Statistical significance *p* < 0.05 (*n* = 3).

Figure 3A shows that benzyl alcohol oxidation was negligible in the absence of Fe_3_O_4_. The 0 mg controls produced only 1% conversion without H_2_O_2_ and 3% conversion with H_2_O_2_ after 24 h, confirming minimal autoxidation and minimal oxidation by H_2_O_2_ alone under these conditions, consistent with previous catalyst-free controls for benzyl alcohol oxidation.^1,61^ Addition of 0.5 mg commercial Fe_3_O_4_ (1.2 mol % relative to benzyl alcohol) increased conversion to 27 ± 1%, while 2 mg (4.9 mol %) produced 72 ± 2% conversion. Further increases in Fe_3_O_4_ loading produced comparatively modest improvements, with conversion reaching 82 ± 1%, at 20 mg (49.4 mol %), indicating diminishing returns beyond 2 mg. This while benzaldehyde selectivity remained relatively consistent across the Fe_3_O_4_ loadings, decreasing only slightly from a maximum of 40 ± 2% at 0.5 mg to 37 ± 2% at 20 mg. As such, benzaldehyde yield increased only from 27.8% at 2 mg to 30.5% at 20 mg (Table S1).

The 2 mg Fe_3_O_4_ loading corresponded to approximately 11.4 mg catalyst/mmol benzyl alcohol, placing it near the lower end of the catalyst-to-substrate ratios represented by previously reported iron oxide-containing systems.^42,43^ These studies achieved moderate to near-complete conversion or benzaldehyde yield using catalyst loadings of approximately 11.4–50 mg/mmol, although the higher-performing systems employed microwave irradiation or engineered multifunctional catalysts under solvent-free conditions. The 72 ± 2% conversion obtained here is therefore encouraging in the context of the comparatively low catalyst loading and unoptimized aqueous conditions employed, although benzaldehyde selectivity was lower than that reported for the optimized systems. In a more compositionally relevant aqueous Fe_3_O_4_ system, unmodified microspheres produced 5.7% conversion with 98.8% benzaldehyde selectivity at 100 °C.^62^ This result demonstrates that unmodified Fe_3_O_4_ can favor selective benzaldehyde formation in water, but with only limited substrate conversion. Against this background, the commercial Fe_3_O_4_ evaluated here achieved substantially greater conversion at a lower temperature, albeit over a considerably longer reaction period and with lower selectivity. This contrasting performance further illustrates the balance between conversion and benzaldehyde selectivity across iron oxide-catalyzed systems. Direct performance ranking remains inappropriate because of differences in catalyst formulation, substrate concentration, oxidant loading, reaction medium, heating method and reaction duration.

Figure 3B shows that magnetosome-catalyzed benzyl alcohol conversion increased with both catalyst loading and reaction time. Addition of 2 mg magnetosomes (1.0 mol % Fe_3_O_4_ equivalent) gave measurable activity, with conversion increasing from 8 ± 4% after 4 h to 12 ± 3% after 8 h and 28 ± 4% after 24 h. Increasing the loading further to 10 mg (5.2 mol % Fe_3_O_4_) enhanced conversion to 64 ± 4% after 24 h, which may reflect greater availability of Fe-containing surface sites and increased H_2_O_2_ activation.^63,64^ However, neither surface-site involvement nor H_2_O_2_ activation was directly examined. Accordingly, mechanistic interpretations should be regarded as hypotheses consistent with the aforementioned Fe_3_O_4_/H_2_O_2_ systems rather than pathways demonstrated by the present data. While conversion did not increase linearly with catalyst mass, potentially indicating incomplete utilization of the additional catalytic material, it did continue to increase at both loadings over the 24 h without reaching an activity plateau. Magnetosomes promoted benzyl alcohol oxidation toward benzaldehyde at both catalyst loadings. The highest benzaldehyde selectivity was observed after 4 and 8 h (43 ± 2%), although selectivity decreased slightly with time. This behavior demonstrates the conversion-selectivity trade-off as magnetosome loading and reaction time increased. As benzaldehyde selectivity did not exceed 43%, more than half of the converted substrate was not assigned to quantified products. No secondary products were reliably identified or quantified, and carbon closure was therefore not achieved. Secondary oxidation products may contribute to this unassigned fraction and reduction of selectivity, but this interpretation remains tentative.^65^

To enable a direct material-level comparison, catalytic performance was assessed at approximately matched Fe_3_O_4_ contents rather than by calculating turnover frequency (TOF). The 10 mg magnetosome catalyst was compared with 2 mg commercial Fe_3_O_4_ at approximately matched Fe_3_O_4_ contents (5.2 versus 4.9 mol %, respectively). After 24 h the commercial Fe_3_O_4_ produced 72 ± 2% conversion and 38 ± 3% selectivity; compared to the 64 ± 4% conversion and 36 ± 5% selectivity seen from 10 mg magnetosomes. A two-sided Welch’s *t*-test not assuming equal variance showed that conversion was significantly higher with commercial Fe_3_O_4_ (*p* = 0.006), whereas the difference in benzaldehyde selectivity was not significant (*p* = 0.171). The corresponding benzaldehyde yields were 27.8% for commercial Fe_3_O_4_ and 22.8% for magnetosomes (Table S1). The lower conversion obtained with magnetosomes could arise from their structure as biogenic, membrane-bound MNPs made of Fe_3_O_4_ presenting constraints not shared by unencapsulated Fe_3_O_4_. Surface modification of Fe_3_O_4_ MNPs can reduce H_2_O_2_-dependent activity by shielding the oxide surface and restricting access to active iron sites, with the extent of inhibition influenced by the properties of the surface coating.^66^ Protein shells and phospholipid bilayers may similarly alter substrate access and transport to encapsulated nanoparticle catalysts.^67,68^ The effect of membrane removal was not evaluated in the present study, as disruption of the magnetosome membrane can cause particle agglomeration and thereby alter the stability and functional properties of magnetosomes.^69^ BET surface-area measurements would therefore be required to determine whether differences in specific surface area contribute to the higher conversion observed with commercial Fe_3_O_4_.

Temperature also affected catalytic performance (Figure 3C). Increasing temperature from 75 to 85 °C enhanced 24 h conversion from 54 ± 3% to 75 ± 11%, consistent with faster oxidation kinetics. At 24 h, Tukey-adjusted comparisons following one-way ANOVA showed that conversion was significantly higher at 85 °C than at 80 °C (*p* = 0.012). Although benzaldehyde selectivity decreased from 42 ± 3% at 75 °C to 30 ± 4% at 85 °C, selectivity at 85 °C was not significantly lower than at 80 °C (*p* = 0.130). Overall, higher temperatures improved conversion while tending to reduce selectivity, a trade-off commonly observed in H_2_O_2_-driven benzylic alcohol oxidation.^70^ Consequently, benzaldehyde yields at 80 and 85 °C were nearly identical at 22.7% and 22.5%, respectively (Table S1). The observed trend may reflect temperature-dependent H_2_O_2_ activation and decomposition at iron sites, as described for Fe_3_O_4_-based oxidation systems.^71,72^ This interpretation remains speculative because radical formation, H_2_O_2_ decomposition, changes in iron oxidation state and iron leaching were not measured directly. A plausible but unverified pathway for the observed catalytic trends involves H_2_O_2_ activation at the Fe_3_O_4_ surface. H_2_O_2_ can adsorb at mixed-valent Fe^2+^/Fe^3+^ sites on Fe_3_O_4_ surfaces.^73^ The presence of surface Fe^2+^ can promote H_2_O_2_ activation and the formation of reactive hydroxyl species.^74^ If formed under the present conditions, these hydroxyl radicals could facilitate the oxidation of benzyl alcohol to benzaldehyde.^62^ Continued oxidation may subsequently produce benzoic acid.^75^ Hydroxyl-radical oxidation can also generate other oxygenated or fragmented products.^76^ Such secondary products could contribute to the reduced benzaldehyde selectivity observed at higher temperatures and the unassigned carbon fraction. However, none was identified or quantified, and the available data do not permit the unassigned carbon to be attributed to any specific compound. Moreover, the present data do not establish whether oxidation proceeds through hydroxyl radicals generated by Fe^2+^/Fe^3+^ cycling, direct surface activation or contributions from dissolved iron species. The identities of the resulting secondary products were not determined in the present study.

Despite the potential constraints imposed by the magnetosome membrane, the observed activity was comparable to exploratory Fe_3_O_4_ MNPs, which have reported 33% conversion under related conditions, albeit with higher selectivity.^14^ When expressed in terms of overall yield (eq 2), this corresponds to 32%, which is comparable to the 22.5% yield obtained here under the most forcing conditions (Table S1). Magnetosomes therefore do not yet match the high selectivity reported for several optimized synthetic catalysts included in Table 1. At this proof-of-concept stage, their principal distinction lies instead in their biological synthesis, measurable activity under unoptimized aqueous conditions and direct magnetic recovery without centrifugation or filtration.

To assess the practical advantage of magnetic recoverability, catalyst recyclability was evaluated over five consecutive cycles under representative conditions (10 mg magnetosomes, 5.2 mol % Fe_3_O_4_, 80 °C). After each run, magnetosomes were recovered using a 1.4 T external magnet, washed with Milli-Q water, and resuspended in fresh reaction solution without centrifugation or filtration. Recyclability performance is presented in Figure 4.

**Figure 4:**
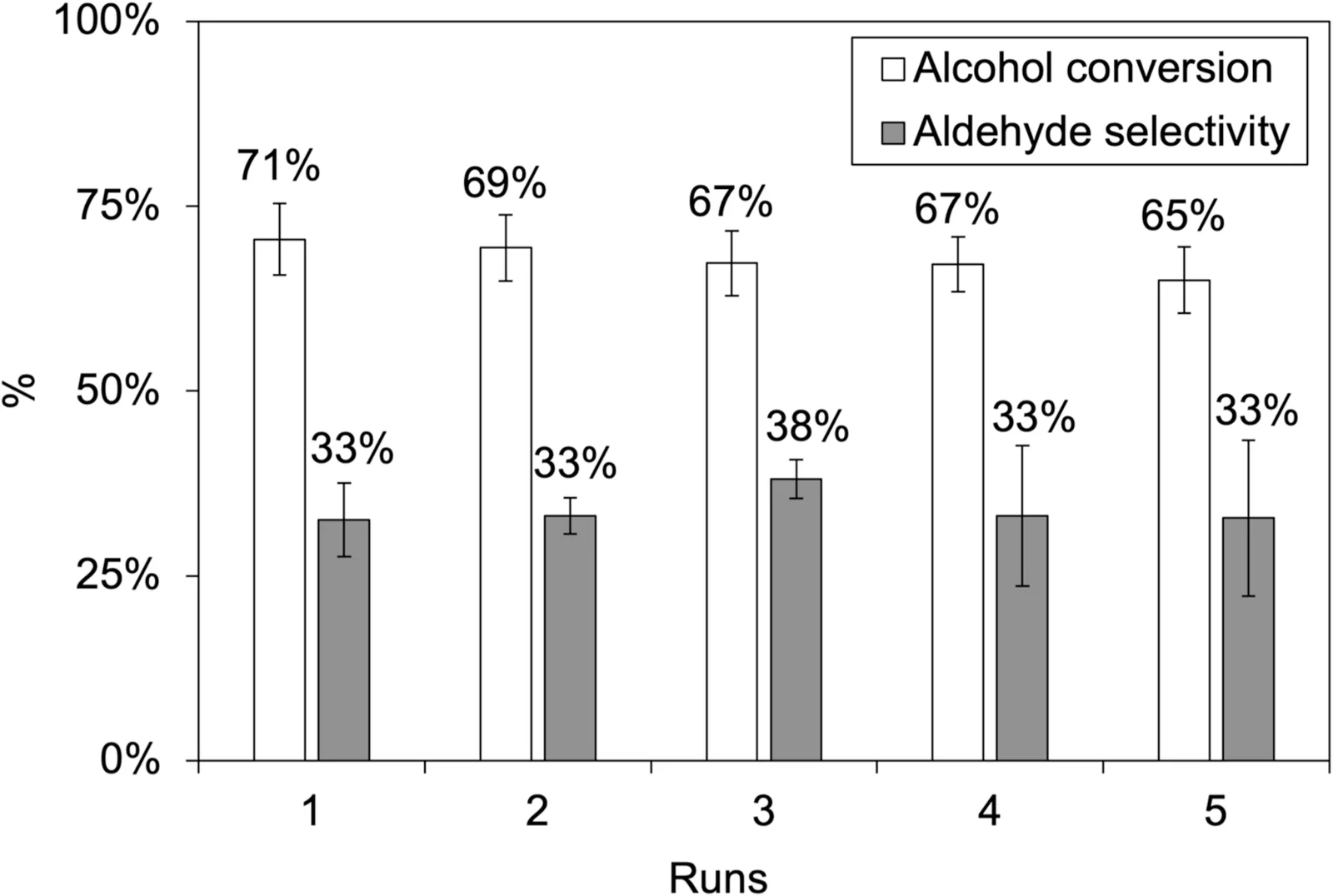
Recyclability of magnetosome catalysts for benzyl alcohol oxidation. Reaction conditions: 25 mM benzyl alcohol, 0.10% (v/v) H_2_O_2_, 10 mg magnetosomes, approximately 5.2 mol % Fe_3_O_4_, 7 mL total volume, 80 °C, 750 rpm, 24 h per run. After each cycle, magnetosomes were magnetically recovered, washed with water, and reused. A one-way repeated-measures ANOVA detected no significant effect of reuse cycle on conversion (*F*(4,8) = 1.88, *p* = 0.207) or benzaldehyde selectivity (*F*(4,8) = 1.68, *p* = 0.247). (*n* = 3).

Figure 4 shows that benzyl alcohol conversion decreased modestly from 71 ± 5% in the first cycle to 65 ± 4% by the fifth cycle, while benzaldehyde selectivity remained comparatively stable at 33–38%, although the associated variability increased from ±2% to ±11% across the five runs. A one-way repeated-measures ANOVA detected no statistically significant effect of reuse cycle on either conversion (*F*(4,8) = 1.88, *p* = 0.207) or benzaldehyde selectivity (*F*(4,8) = 1.68, *p* = 0.247). Although the initial 71% conversion was slightly higher than observed previously under identical conditions, it remained within the same overall performance range, with no statistically significant difference detected by *t*-test. This behavior is consistent with reports of magnetically recoverable heterogeneous catalysts showing only slight decreases in activity upon reuse.^77^ In wider iron oxide-based systems, gradual deactivation can arise from physicochemical changes during operation, including oxidation-state shifts to less active iron oxide phases, surface restructuring, aggregation, and loss of accessible active sites.^78^

The modest decline in conversion may reflect practical catalyst losses during recovery, iron leaching, or both, rather than intrinsic deactivation alone. During manual magnetic separation and washing, small quantities of magnetosomes may have been lost between cycles, reducing the effective catalyst mass in later reactions. Catalyst loss during washing and magnetic recovery has previously been identified as a contributor to apparent activity decay in magnetically recoverable catalyst-reuse studies.^79^ Any iron dissolved during a reaction would similarly have been removed when the liquid reaction mixture was completely replaced between cycles. As leached iron could contribute to homogeneous Fenton-type activity within each reaction, progressive depletion of leachable iron from the recovered magnetosomes, or reduced iron release during successive cycles, could contribute to the lower conversion observed in later runs. Relationships between iron leaching, homogeneous activity and catalyst deactivation during repeated use have previously been reported for Fe_3_O_4_/H_2_O_2_ systems.^80^ Variations in magnetic recovery and iron dissolution could also contribute to run-to-run variability, consistent with the larger selectivity error observed in the fifth cycle.^81^ However, because iron concentrations in the reaction supernatants were not measured, the occurrence and extent of iron leaching remain unknown. The present study therefore cannot distinguish among heterogeneous surface-mediated catalysis, homogeneous Fenton-type catalysis by dissolved iron, or a combination of these mechanisms; supernatant iron quantification and catalyst-removal experiments would be required to resolve their relative contributions. The retention of activity and relatively stable selectivity over five cycles demonstrates that the magnetosome material can be magnetically recovered and reused without a substantial loss of overall reaction performance, but neither establishes an exclusively heterogeneous pathway nor confirms that the recovered magnetosomes remained structurally and chemically unchanged. Consequently, postreaction characterization by XRD, TEM, FTIR, and VSM would be required to assess possible phase transformation, particle aggregation, membrane degradation and changes in magnetic properties.

Taken together, the findings of this study position magnetosomes as a distinct rather than directly competitive catalytic system relative to the well-defined catalysts summarized in Table 1. Higher conversions and selectivities have been reported using engineered supported catalysts, mixed-metal ferrites and noble-metal systems. However, these frequently rely on organic or solvent-free media, microwave heating, pressurized O_2_, *tert*-butyl hydroperoxide, noble metals or specifically fabricated catalyst supports.^1,2,6–8,18^ Direct numerical comparison is limited by substantial differences in catalyst composition, substrate, oxidant, solvent and reaction configuration. The novelty of the present system lies not in outperforming these optimized catalysts, but in demonstrating that biologically synthesized, membrane-bound Fe_3_O_4_ MNPs exhibit oxidation activity while retaining the inherent magnetic recoverability of the material. However, the relative contributions of surface-mediated and dissolved-iron pathways remain unresolved. Their biogenic formation and lack of noble-metal incorporation or a deliberately engineered inorganic support distinguish magnetosomes from many of the aforementioned synthetic systems. This study therefore establishes the catalytic feasibility of a distinctive biogenic material. Nevertheless, considerable work remains to improve reaction efficiency and product selectivity, establish catalyst stability during reuse, explore a broader substrate scope and determine whether the system offers an overall sustainability advantage. These limitations are addressed in full within the next section.

## 4 Scope and Limitations of the Study

This study was designed as an initial proof of concept to determine whether purified magnetosomes exhibit measurable activity in benzyl alcohol oxidation and can subsequently be magnetically recovered and reused. The physicochemical analyses confirmed the presence of crystalline Fe_3_O_4_, canonical magnetosome morphology and magnetic behavior suitable for recovery. However, full structural and activity characterisations were not performed. In particular, specific surface area and porosity were not determined by BET analysis, while membrane composition and Fe_3_O_4_ surface accessibility were not characterized. In addition, the composition of the TGA residue was not independently confirmed and may not correspond exclusively to Fe_3_O_4_. The 21 wt % residue was therefore used only as an Fe_3_O_4_-equivalent value for approximate mass normalization. This comparison does not account for potential differences in accessible surface area, membrane coverage or active-site density between magnetosomes and commercial Fe_3_O_4_. Consequently, turnover frequency (TOF) was not calculated because the number of accessible active sites could not be reliably defined, and the experiments were not designed to determine initial reaction rates.

The catalytic testing was deliberately limited to a maximum of 24 h and 85 °C, as the aim was to demonstrate proof-of-concept activity and responsiveness to these variables rather than exhaustively optimize the reaction. Longer-duration and higher-temperature experiments will be required to define the wider operating window and determine whether conversion increases further or reaches an optimum. Product distribution was not fully characterized, and carbon closure was not achieved. Given benzaldehyde selectivities of approximately 30–43%, an estimated 57–70% of the converted substrate was not assigned to quantified products. Accordingly, the mechanistic interpretations should be regarded as provisional hypotheses rather than pathways established by direct experimental evidence. Further work should include identification and quantification of secondary products, establishment of a complete carbon balance and measurement of iron leaching. Radical-scavenging and spectroscopic studies, together with catalyst-removal experiments, would be required to distinguish hydroxyl-radical pathways involving Fe^2+^/Fe^3+^ cycling from direct surface activation and contributions from dissolved iron species. These analyses would help distinguish among the proposed pathways and guide further development of the system. Similarly, substrate scope was not systematically evaluated beyond the model benzyl alcohol reaction, and future studies should assess structurally diverse alcohols and other oxidation substrates under substrate-specific conditions.

Finally, the five-cycle experiment demonstrates practical magnetic recovery and retention of catalytic activity but does not establish that the magnetosomes remained structurally and chemically unchanged during reuse. The recovered catalyst was not characterized by XRD, TEM, FTIR, VSM or BET analysis. Consequently, the modest change in performance cannot be attributed specifically to catalyst loss, aggregation, phase transformation, membrane degradation, altered surface properties or iron leaching. Residual H_2_O_2_ should also be quantified at the reaction end point to distinguish oxidant consumption through substrate oxidation from competing peroxide decomposition associated with solid or dissolved iron species.

These limitations do not alter the central proof-of-concept observation that biogenic magnetosomes promote measurable benzyl alcohol conversion and retain activity following magnetic recovery. However, because iron leaching was not measured, the present study cannot distinguish between heterogeneous surface-mediated catalysis, homogeneous Fenton-type catalysis by dissolved iron, or a combination of these mechanisms. Instead, these limitations define the surface, leaching, product, mechanistic and postreaction analyses required to distinguish among the proposed catalytic pathways and determine whether the biological membrane or magnetosome chain architecture provides advantages over conventional Fe_3_O_4_ catalysts.

## 5 Conclusion

Overall, this proof-of-concept study establishes purified biogenic magnetosomes as magnetically recoverable catalyst materials for benzyl alcohol oxidation using H_2_O_2_. Characterization confirmed a crystalline Fe_3_O_4_ core, canonical magnetosome morphology and strong remanent magnetic polarization suitable for direct magnetic separation. Under unoptimized aqueous conditions, 10 mg magnetosomes produced 64 ± 4% conversion and approximately 36 ± 5% benzaldehyde selectivity after 24 h. At approximately matched Fe_3_O_4_ content, 2 mg commercial Fe_3_O_4_ produced 72 ± 2% conversion and approximately 38 ± 3% selectivity, demonstrating broadly comparable performance despite the membrane-bound structure of the biogenic material. However, this comparison remains approximate because BET surface-area measurements were not performed and the relative accessibility of the magnetite surfaces could not be established. Magnetosome-catalyzed conversion increased to 75 ± 11% at 85 °C, while the highest benzaldehyde selectivity was 43 ± 2% under milder conditions. Magnetosomes were also recovered and reused over five cycles, with conversion decreasing only modestly from 71 ± 5% to 65 ± 4%.

These findings provide the first evidence that biogenic magnetosomes can facilitate benzyl alcohol oxidation while retaining the practical advantage of magnetic recovery. Their significance at this stage lies in establishing a new membrane-bound, biogenic catalytic platform rather than outperforming optimized synthetic catalysts. However, further leaching, product and spent-catalyst analyses are required to determine the operative mechanism, confirm catalyst stability and establish whether the native membrane and chain architecture can be exploited to improve catalytic performance.

## Supplementary Material



## Notes

Benzyl alcohol oxidation reactions, including reaction conditions and corresponding conversion, selectivity, and yield data (PDF)
