# Supplementary material for: Biogenic Magnetosomes as Magnetically Recoverable Catalysts for Benzyl Alcohol Oxidation
Source: ACS Omega. 2026 Oct 9;11(41):63744–54. doi: 10.1021/acsomega.6c07960 (PMC13689274; doi:10.1021/acsomega.6c07960)
Supplement: Supplementary file 1 [file ao6c07960_si_001.pdf]

Supporting Information for:

Biogenic Magnetosomes as Magnetically Recoverable Catalysts for Benzyl Alcohol Oxidation

Josh Bond[1,2], Marcia Mendes de Carvalho[1], Amin Osatiashtiani[3], Tim William Overton[4], Oliver Brooks[5], Vesna Najdanovic-Visak[1] & Alfred Fernandez-Castane*[1,2]

***Corresponding author**

[1] Energy and Bioproducts Research Institute, Aston University, Aston Triangle, Birmingham B4 7ET, United Kingdom

[2] Aston Institute for Membrane Excellence, Aston University, Aston Triangle, Birmingham B4 7ET, United Kingdom

[3] School of Chemical and Process Engineering, University of Leeds, Leeds LS2 9JT, United Kingdom

[4] School of Chemical Engineering, University of Birmingham, Edgbaston, Birmingham B15 2TT, United Kingdom

[5] School of Metallurgy and Materials, University of Birmingham, Edgbaston, Birmingham B15 2TT, United Kingdom

Table S1 – Summary the reaction conditions presented in Figure 3. Mean benzyl alcohol conversion and benzaldehyde selectivity, with benzaldehyde yield calculated from mean values using equations 1-3 respectively. (*n* = 3).

	**Variable**	**Time (h)**	**Conversion (%)**	**Selectivity (%)**	**Yield (%)**
**Figure 3A** – Effect of Fe_3O_4 loading	0 mg + no H_2O_2	24	1.3	2.0	0.0
	0 mg	24	3.2	16.1	0.5
	0.5 mg	24	26.9	40.2	10.8
	2 mg	24	72.4	38.4	27.8
	10 mg	24	78.5	38.3	30.1
	20 mg	24	82.4	37.0	30.5
**Figure 3B** – Effect of magnetosome loading	2 mg	4	7.6	42.9	3.2
		8	11.6	42.6	4.9
		24	27.8	34.8	9.7
	10 mg	4	14.9	37.1	5.5
		8	26.4	37.4	9.9
		24	63.5	36.0	22.8
**Figure 3C** – Effect of reaction temperature on magnetosome catalytic function	75°C	8	14.3	39.0	5.6
		24	53.9	41.7	22.5
	80°C	8	24.7	35.9	8.9
		24	65.0	34.9	22.7
	85°C	8	29.8	25.1	7.5
		24	75.0	30.0	22.5
